# The genome sequence of the pink-footed goose, *Anser brachyrhynchus *Baillon, 1834

**DOI:** 10.12688/wellcomeopenres.23194.1

**Published:** 2024-10-18

**Authors:** Rosa Lopez Colom, Michelle O’Brien, Natural History Museum Genome Acquisition Lab, Darwin Tree of Life Barcoding collective, Wellcome Sanger Institute Tree of Life Management, Samples and Laboratory team, Wellcome Sanger Institute Scientific Operations: Sequencing Operations, Wellcome Sanger Institute Tree of Life Core Informatics team, Tree of Life Core Informatics collective, Darwin Tree of Life Consortium

**Affiliations:** 1 Wildfowl & Wetlands Trust, Slimbridge, Gloucestershire, England, GL2 7BT, UK

**Keywords:** Anser brachyrhynchus, pink-footed goose, genome sequence, chromosomal, Anseriformes

## Abstract

We present a genome assembly from a female pink-footed goose, *Anser brachyrhynchus* (Chordata; Aves; Anseriformes; Anatidae). The genome sequence spans 1,287.30 megabases. Most of the assembly is scaffolded into 41 chromosomal pseudomolecules, including the W and Z sex chromosomes. The mitochondrial genome has also been assembled and is 16.74 kilobases in length.

## Species taxonomy

Eukaryota; Opisthokonta; Metazoa; Eumetazoa; Bilateria; Deuterostomia; Chordata; Craniata; Vertebrata; Gnathostomata; Teleostomi; Euteleostomi; Sarcopterygii; Dipnotetrapodomorpha; Tetrapoda; Amniota; Sauropsida; Sauria; Archelosauria; Archosauria; Dinosauria; Saurischia; Theropoda; Coelurosauria; Aves; Neognathae; Galloanserae; Anseriformes; Anatidae; Anserinae; *Anser*; *Anser brachyrhynchus* Baillon, 1834 (NCBI:txid132585).

## Background

The pink-footed goose ( *Anser brachyrhynchus*) is commonly observed in the UK during the winter months. This species is distinguished by its overall grey plumage, feathers edged in white, dark head and neck, pale chest, and a small dark bill with a pink band. The limbs vary in colour from pale to deep pink, as reflected in its common name ( Hume, 2014). There is no sexual dimorphism in plumage patterns. However, males tend to have an average weight of 2.6 kg and females 2.3 kg, while both sexes have an average length of 75 cm and width of 165 cm ( Notario Gómez, 2009). Pink-footed geese are similar in appearance to their close relative, the greylag goose ( *Anser anser*), but are slightly smaller in size, with shorter necks and wingspans ranging from 137 cm to 161 cm ( Hume, 2014). They are easily identifiable by their characteristic vocalisations, which resemble a ‘wink wink’ sound, particularly when gathered in large, noisy colonies.

Pink-footed geese are highly gregarious, and migrate in large flocks across the East Atlantic flyway during the early spring months (breeding season), travelling to Iceland, Greenland, and Svalbard, where they are typically found nesting in central highland regions ( Hilmarsson, 2021). This is in contrast to their wintering habits in northwestern Europe, where they predominantly graze and roost in agricultural fields and large estuaries.

Migration routes in pink-footed geese are culturally inherited within flocks. However, there is evidence that colonies have, over the years, begun to follow new routes and adopt different stopover sites. They have also established breeding grounds beyond their historically known locations ( Madsen *et al.*, 2023). In light of these changes, genomic studies are invaluable for tracing genetic dispersal and understanding the formation of new colonies by younger geese as they adapt to shifting habitat conditions and environmental changes.

Although classified as a species of ‘Least Concern’ in IUCN’s Red list ( BirdLife International, 2016), in the UK it is considered to have an Amber conservation status and is further protected under the Wildlife and Countryside Act, 1981 ( The Wildlife Trusts, 2020).

Here we present a chromosomally complete genome sequence for *Anser brachyrhynchus*, based on a female individual goose found deceased in Lancashire. 

## Genome sequence report

**Figure 1.**
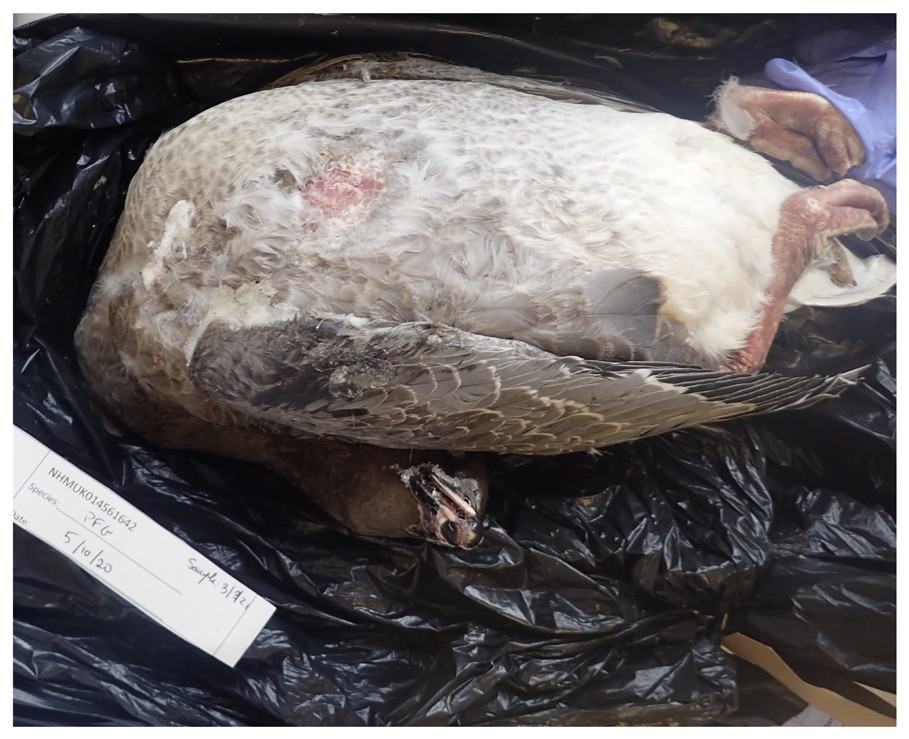
Photograph of the *Anser brachyrhynchus* (bAnsBra1) specimen used for genome sequencing.

The genome of an adult female *Anser brachyrhynchus* ( Figure 1) was sequenced using Pacific Biosciences single-molecule HiFi long reads, generating a total of 48.77 Gb (gigabases) from 4.78 million reads, providing approximately 38-fold coverage. Primary assembly contigs were scaffolded with chromosome conformation Hi-C data, which produced 98.03 Gb from 649.21 million reads, yielding an approximate coverage of 76-fold. Specimen and sequencing information is summarised in Table 1.

**Table 1.** Specimen and sequencing data for *Anser brachyrhynchus*.

Project information			
**Study title**	*Anser brachyrhynchus* (pink-footed goose)		
**Umbrella BioProject**	PRJEB68284		
**BioSample**	SAMEA112468040		
**NCBI taxonomy ID**	132585		
Specimen information			
**Technology**	**ToLID**	**BioSample ** **accession**	**Organism ** **part**
**PacBio long read sequencing**	bAnsBra1	SAMEA112468100	muscle
**Hi-C sequencing**	bAnsBra1	SAMEA112468100	muscle
**RNA sequencing**	bAnsBra1	SAMEA112468100	muscle
Sequencing information			
**Platform**	**Run ** **accession**	**Read count**	**Base ** **count (Gb)**
**Hi-C Illumina NovaSeq 6000**	ERR12259840	6.49e+08	98.03
**PacBio Sequel IIe**	ERR12257414	2.24e+06	22.83
**PacBio Sequel IIe**	ERR12257415	2.54e+06	25.94
**RNA Illumina NovaSeq 6000**	ERR12321240	7.07e+07	10.67

**Figure 2.**
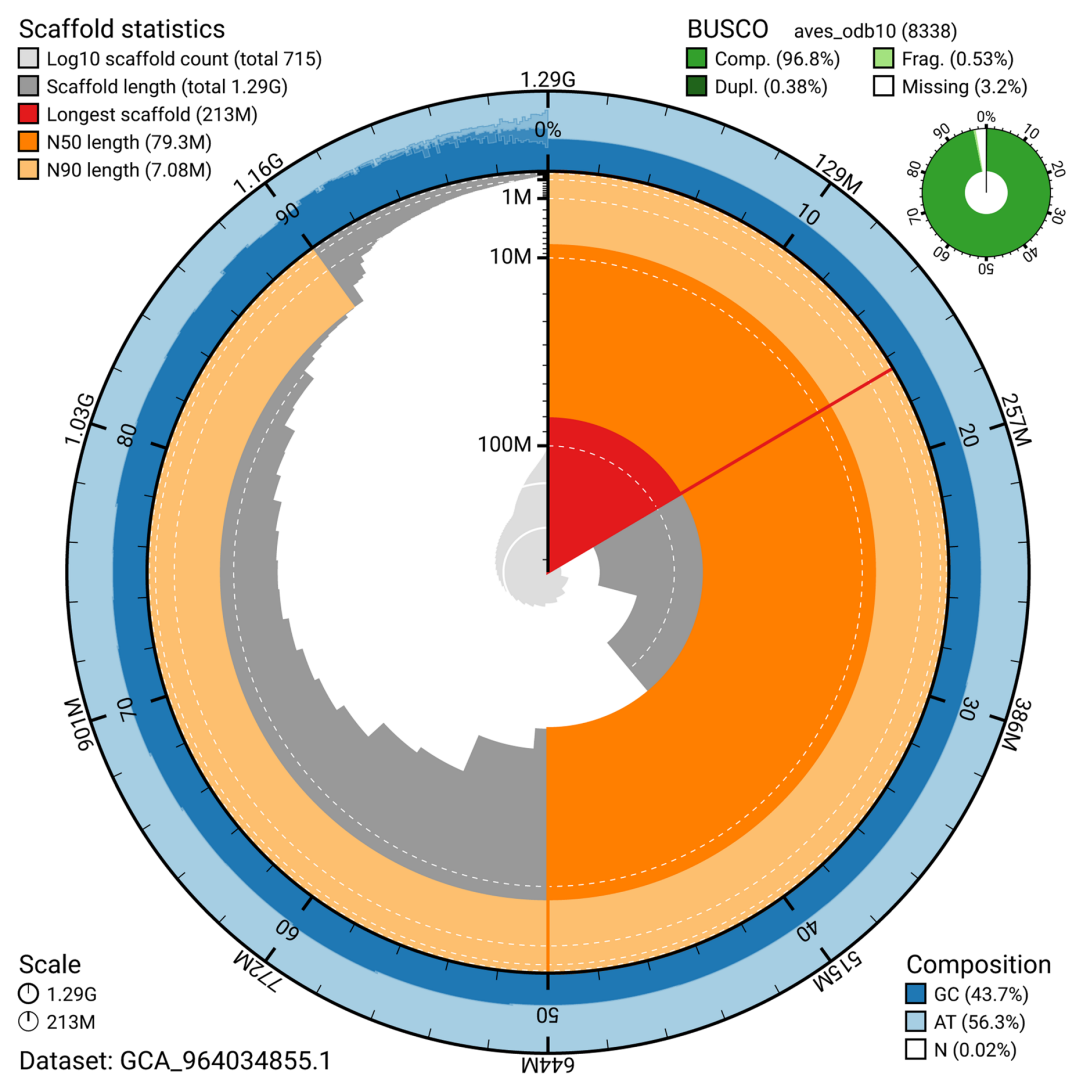
Genome assembly of *Anser brachyrhynchus*, bAnsBra1.1: metrics. The BlobToolKit snail plot shows N50 metrics and BUSCO gene completeness. The main plot is divided into 1,000 size-ordered bins around the circumference with each bin representing 0.1% of the 1,287,337,719 bp assembly. The distribution of scaffold lengths is shown in dark grey with the plot radius scaled to the longest scaffold present in the assembly (213,089,697 bp, shown in red). Orange and pale-orange arcs show the N50 and N90 scaffold lengths (79,251,119 and 7,082,356 bp), respectively. The pale grey spiral shows the cumulative scaffold count on a log scale with white scale lines showing successive orders of magnitude. The blue and pale-blue area around the outside of the plot shows the distribution of GC, AT and N percentages in the same bins as the inner plot. A summary of complete, fragmented, duplicated and missing BUSCO genes in the aves_odb10 set is shown in the top right. An interactive version of this figure is available at https://blobtoolkit.genomehubs.org/view/GCA_964034855.1/dataset/GCA_964034855.1/snail.

**Figure 3.**
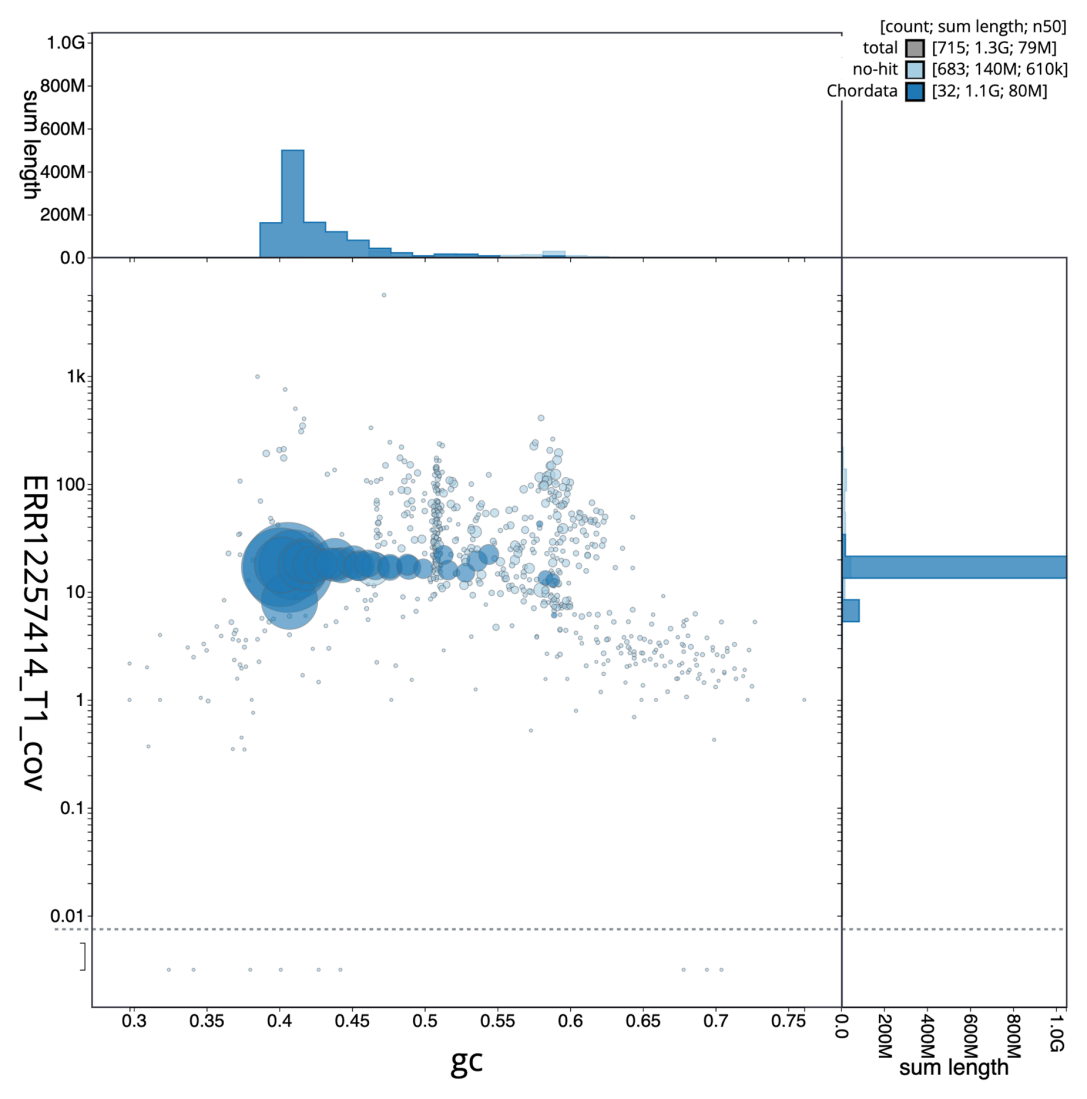
Blob plot of base coverage in ERR12257414 against GC proportion for sequences in assembly GCA_964034855.1. Sequences are coloured by phylum. Circles are sized in proportion to sequence length. Histograms show the distribution of sequence length sum along each axis. An interactive version of this figure is available at https://blobtoolkit.genomehubs.org/view/GCA_964034855.1/dataset/GCA_964034855.1/blob.

**Figure 4.**
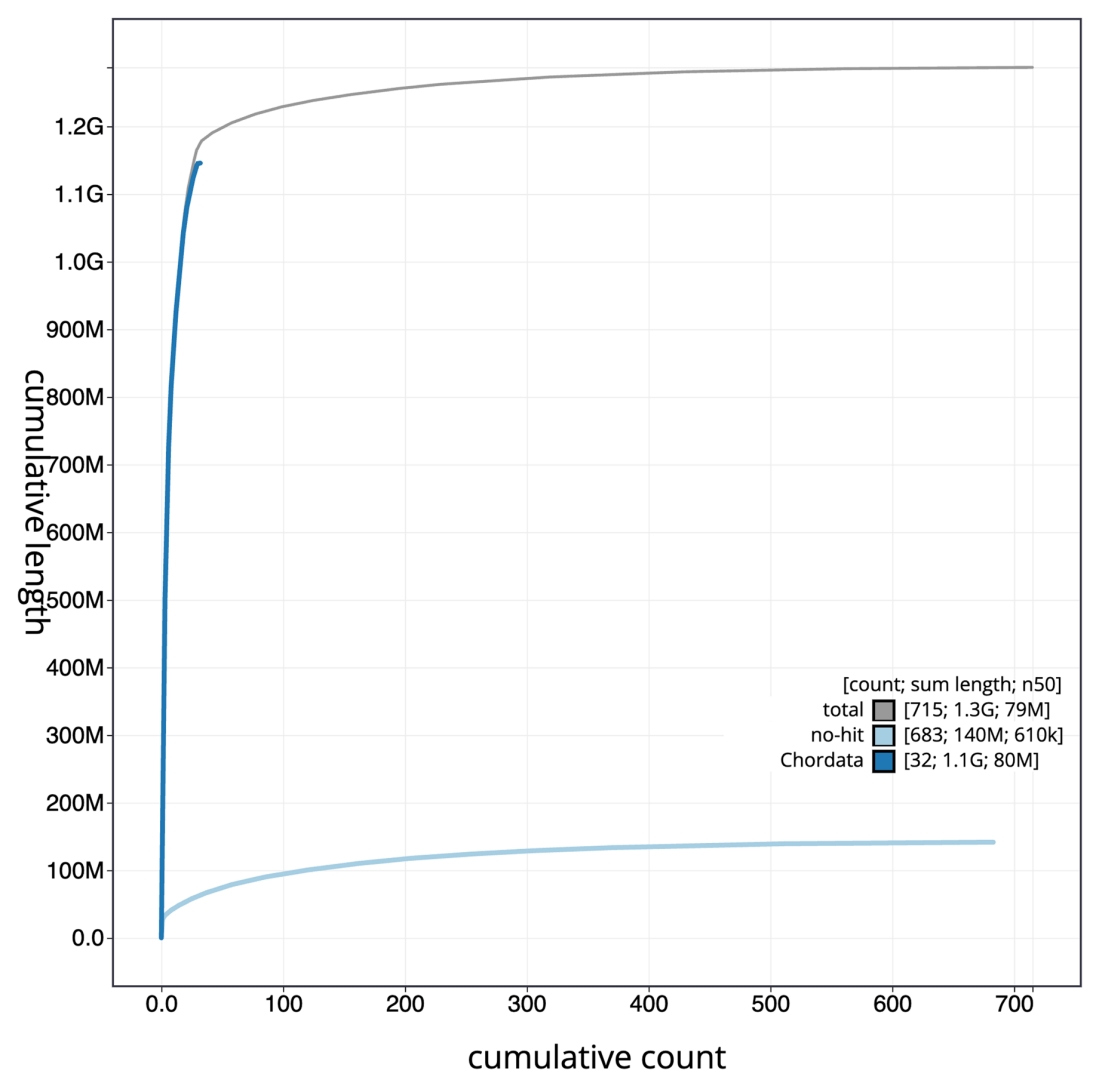
Genome assembly of *Anser brachyrhynchus* bAnsBra1.1: BlobToolKit cumulative sequence plot. The grey line shows cumulative length for all sequences. Coloured lines show cumulative lengths of sequences assigned to each phylum using the buscogenes taxrule. An interactive version of this figure is available at https://blobtoolkit.genomehubs.org/view/GCA_964034855.1/dataset/GCA_964034855.1/cumulative.

**Figure 5.**
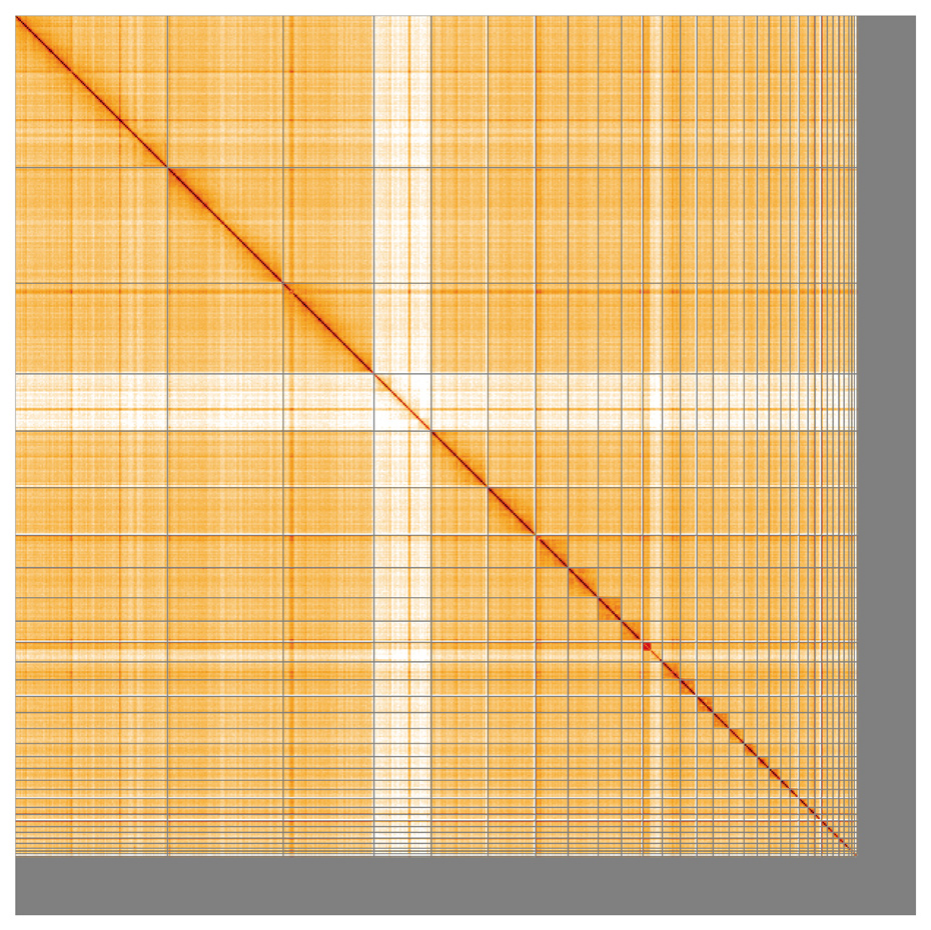
Genome assembly of *Anser brachyrhynchus* bAnsBra1.1: Hi-C contact map of the bAnsBra1.1 assembly, visualised using HiGlass. Chromosomes are shown in order of size from left to right and top to bottom. An interactive version of this figure may be viewed at https://genome-note-higlass.tol.sanger.ac.uk/l/?d=VHwM1bk0RG6VnG7ZhbJfhQ.

Manual assembly curation corrected 76 missing joins or mis-joins, reducing the scaffold number by 6.17%, and increasing the scaffold N50 by 19.06%. The final assembly has a total length of 1,287.30 Mb in 714 sequence scaffolds, with 1,137 gaps, and a scaffold N50 of 79.3 Mb ( Table 2). The snail plot in Figure 2 provides a summary of the assembly statistics, while the distribution of assembly scaffolds on GC proportion and coverage is shown in Figure 3. The cumulative assembly plot in Figure 4 shows curves for subsets of scaffolds assigned to different phyla. Most (91.45%) of the assembly sequence was assigned to 41 chromosomal-level scaffolds, representing 39 autosomes and the W and Z sex chromosomes. Chromosome-scale scaffolds confirmed by the Hi-C data are named in order of size ( Figure 5; Table 3). Note that the PAR region is collapsed and is represented on Chromosome W only.

**Table 2.** Genome assembly data for *Anser brachyrhynchus*, bAnsBra1.1.

Genome assembly		
Assembly name	bAnsBra1.1	
Assembly accession	GCA_964034855.1	
*Accession of alternate * *haplotype*	*GCA_964034845.1*	
Span (Mb)	1,287.30	
Number of contigs	1,852	
Contig N50 length (Mb)	2.0	
Number of scaffolds	714	
Scaffold N50 length (Mb)	79.3	
Longest scaffold (Mb)	213.09	
Assembly metrics *		*Benchmark*
Consensus quality (QV)	58.2	*≥ 50*
*k*-mer completeness	100.0%	*≥ 95%*
BUSCO **	C:96.9%[S:96.5%,D:0.4%], F:0.5%,M:2.6%,n:8,338	*C ≥ 95%*
Percentage of assembly mapped to chromosomes	91.45%	*≥ 95%*
Sex chromosomes	WZ	*localised homologous pairs*
Organelles	Mitochondrial genome: 16.74 kb	*complete single alleles*

* Assembly metric benchmarks are adapted from column VGP-2020 of “Table 1: Proposed standards and metrics for defining genome assembly quality” from Rhie *et al.* (2021). ** BUSCO scores based on the vertebrata_odb10 BUSCO set using version 5.4.3. C = complete [S = single copy, D = duplicated], F = fragmented, M = missing, n = number of orthologues in comparison. A full set of BUSCO scores is available at https://blobtoolkit.genomehubs.org/view/Anser_brachyrhynchus/dataset/GCA_964034855.1/busco.

**Table 3.** Chromosomal pseudomolecules in the genome assembly of *Anser brachyrhynchus*, bAnsBra1.

INSDC accession	Name	Length (Mb)	GC%
OZ035886.1	1	213.09	40.5
OZ035887.1	2	161.66	40.0
OZ035888.1	3	126.59	41.0
OZ035890.1	4	79.25	40.0
OZ035891.1	5	66.56	41.5
OZ035892.1	6	45.34	44.0
OZ035893.1	7	42.1	41.5
OZ035894.1	8	32.9	42.0
OZ035895.1	9	29.38	44.5
OZ035897.1	10	25.18	45.0
OZ035898.1	11	23.08	44.0
OZ035899.1	12	22.68	43.0
OZ035900.1	13	22.08	43.5
OZ035901.1	14	20.96	45.5
OZ035902.1	15	18.23	45.5
OZ035903.1	16	16.75	46.5
OZ035904.1	17	16.45	46.0
OZ035905.1	18	13.17	47.5
OZ035906.1	19	12.61	49.0
OZ035907.1	20	12.58	47.5
OZ035908.1	21	9.59	49.0
OZ035909.1	22	8.79	53.5
OZ035910.1	23	8.3	54.5
OZ035911.1	24	8.04	51.5
OZ035912.1	25	7.93	50.0
OZ035913.1	26	7.22	51.5
OZ035914.1	27	7.08	53.0
OZ035915.1	28	3.96	58.5
OZ035916.1	29	3.44	59.0
OZ035917.1	30	1.08	54.0
OZ035918.1	31	0.96	51.0
OZ035919.1	32	0.46	59.5
OZ035920.1	33	0.4	51.0
OZ035921.1	34	0.35	55.0
OZ035922.1	35	0.34	59.0
OZ035923.1	36	0.23	59.0
OZ035924.1	37	0.23	61.0
OZ035925.1	38	0.19	56.5
OZ035926.1	39	0.19	54.5
OZ035896.1	W	27.52	46.5
OZ035889.1	Z	80.17	40.5
OZ035927.1	MT	0.02	47.0

While not fully phased, the assembly deposited is of one haplotype. Contigs corresponding to the second haplotype have also been deposited. The mitochondrial genome was also assembled and can be found as a contig within the multifasta file of the genome submission.

The estimated Quality Value (QV) of the final assembly is 58.2 with *k*-mer completeness of 100.0%, and the assembly has a BUSCO v5.4.3 completeness of 96.9% (single = 96.5%, duplicated = 0.4%), using the vertebrata_odb10 reference set ( *n* = 8,338).

Metadata for specimens, BOLD barcode results, spectra estimates, sequencing runs, contaminants and pre-curation assembly statistics are given at https://links.tol.sanger.ac.uk/species/132585.

## Methods

### Sample acquisition

Several small samples of the pectoral muscle were collected from a deceased female pink-footed goose ( *Anser brachyrhynchus*), identified as specimen NHMUK014561642 (ToLID bAnsBra1). This waterfowl was collected in Lancashire in October 2020. Although PCR testing returned a negative result for highly pathogenic avian influenza (HPAI), the specimen was collected shortly before the onset of mortalities in the area caused by the HPAI strain H5N8, which had a significant population-level impact on several goose species across the UK during that winter season. The specimen was collected and identified by Rosa Lopez Colom (Wildfowl & Wetlands Trust) and preserved at –20 °C. Several small samples of muscle tissue were taken and stored at –80 °C.

The initial identification was verified by an additional DNA barcoding process according to the framework developed by Twyford *et al.* (2024). A small sample was dissected from the specimens and stored in ethanol, while the remaining parts were shipped on dry ice to the Wellcome Sanger Institute (WSI). The tissue was lysed, the COI marker region was amplified by PCR, and amplicons were sequenced and compared to the BOLD database, confirming the species identification ( Crowley *et al.*, 2023). Following whole genome sequence generation, the relevant DNA barcode region was also used alongside the initial barcoding data for sample tracking at the WSI ( Twyford *et al.*, 2024). The standard operating procedures for Darwin Tree of Life barcoding have been deposited on protocols.io ( Beasley *et al.*, 2023).

### Nucleic acid extraction

The workflow for high molecular weight (HMW) DNA extraction at the WSI Tree of Life Core Laboratory includes a sequence of core procedures: sample preparation and homogenisation, DNA extraction, fragmentation and purification. Detailed protocols are available on protocols.io ( Denton *et al.*, 2023). The bAnsBra1 sample was prepared for DNA extraction by weighing and dissecting it on dry ice ( Jay *et al.*, 2023).

For sample homogenisation, muscle tissue was cryogenically disrupted using the Covaris cryoPREP ^®^ Automated Dry Pulverizer ( Narváez-Gómez *et al.*, 2023). HMW DNA was extracted in the WSI Scientific Operations core using the Automated MagAttract v2 protocol ( Oatley *et al.*, 2023). The DNA was sheared into an average fragment size of 12–20 kb in a Megaruptor 3 system ( Bates *et al.*, 2023). Sheared DNA was purified by solid-phase reversible immobilisation, using AMPure PB beads to eliminate shorter fragments and concentrate the DNA ( Strickland *et al.*, 2023). The concentration of the sheared and purified DNA was assessed using a Nanodrop spectrophotometer and Qubit Fluorometer using the Qubit dsDNA High Sensitivity Assay kit. Fragment size distribution was evaluated by running the sample on the FemtoPulse system.

RNA was extracted from muscle tissue of bAnsBra1 in the Tree of Life Laboratory at the WSI using the RNA Extraction: Automated MagMax™ *mir*Vana protocol ( do Amaral *et al.*, 2023). The RNA concentration was assessed using a Nanodrop spectrophotometer and a Qubit Fluorometer using the Qubit RNA Broad-Range Assay kit. Analysis of the integrity of the RNA was done using the Agilent RNA 6000 Pico Kit and Eukaryotic Total RNA assay.

### Hi-C preparation

Muscle tissue of the bAnsBra1 sample was processed at the WSI Scientific Operations core, using the Arima-HiC v2 kit. In brief, frozen tissue (stored at –80 °C) was fixed, and the DNA crosslinked using a TC buffer with 22% formaldehyde concentration. After crosslinking the tissue was homogenised using the Diagnocine Power Masher-II and BioMasher-II tubes and pestles. Following the kit manufacturer's instructions, crosslinked DNA was digested using a restriction enzyme master mix. The 5’-overhangs were then filled in and labelled with biotinylated nucleotides and proximally ligated. An overnight incubation was carried out for enzymes to digest remaining proteins and for crosslinks to reverse. A clean up was performed with SPRIselect beads prior to library preparation.

### Library preparation and sequencing

Library preparation and sequencing were performed at the WSI Scientific Operations core. Libraries were prepared using the PacBio Express Template Preparation Kit v2.0 (Pacific Biosciences, California, USA) as per the manufacturer's instructions. The kit includes the reagents required for removal of single-strand overhangs, DNA damage repair, end repair/A-tailing, adapter ligation, and nuclease treatment. Library preparation also included a library purification step using AMPure PB beads (Pacific Biosciences, California, USA) and size selection step to remove templates <3kb using AMPure PB modified SPRI. DNA concentration was quantified using the Qubit Fluorometer v2.0 and Qubit HS Assay Kit and the final library fragment size analysis was carried out using the Agilent Femto Pulse Automated Pulsed Field CE Instrument and gDNA 165kb gDNA and 55kb BAC analysis kit.

Poly(A) RNA-Seq libraries were constructed using the NEB Ultra II RNA Library Prep kit, following the manufacturer’s instructions. RNA sequencing was performed on the Illumina NovaSeq 6000 instrument.

### Sequel IIe sequencing

Samples were sequenced using the Sequel IIe system (Pacific Biosciences, California, USA). The concentration of the library loaded onto the Sequel IIe was between 40 - 135 pM. The SMRT link software, a PacBio web-based end-to-end workflow manager, was used to set-up and monitor the run, as well as perform primary and secondary analysis of the data upon completion.

For Hi-C library preparation, DNA was fragmented to a size of 400 to 600 bp using a Covaris E220 sonicator. The DNA was then enriched, barcoded, and amplified using the NEBNext Ultra II DNA Library Prep Kit following manufacturers’ instructions. The Hi-C sequencing was performed using paired-end sequencing with a read length of 150 bp on an Illumina NovaSeq 6000.

### Genome assembly, curation and evaluation

***Assembly***

The HiFi reads were first assembled using Hifiasm ( Cheng *et al.*, 2021) with the --primary option. Haplotypic duplications were identified and removed using purge_dups ( Guan *et al.*, 2020). The Hi-C reads were mapped to the primary contigs using bwa-mem2 ( Vasimuddin *et al.*, 2019). The contigs were further scaffolded using the provided Hi-C data ( Rao *et al.*, 2014) in YaHS ( Zhou *et al.*, 2023) using the --break option. The scaffolded assemblies were evaluated using Gfastats ( Formenti *et al.*, 2022), BUSCO ( Manni *et al.*, 2021) and MERQURY.FK ( Rhie *et al.*, 2020).

The mitochondrial genome was assembled using MitoHiFi ( Uliano-Silva *et al.*, 2023), which runs MitoFinder ( Allio *et al.*, 2020) and uses these annotations to select the final mitochondrial contig and to ensure the general quality of the sequence.

***Assembly curation***

The assembly was decontaminated using the Assembly Screen for Cobionts and Contaminants (ASCC) pipeline (article in preparation). Flat files and maps used in curation were generated in TreeVal ( Pointon *et al.*, 2023). Manual curation was primarily conducted using PretextView ( Harry, 2022), with additional insights provided by JBrowse2 ( Diesh *et al.*, 2023) and HiGlass ( Kerpedjiev *et al.*, 2018). Scaffolds were visually inspected and corrected as described by Howe *et al.* (2021). Any identified contamination, missed joins, and mis-joins were corrected, and duplicate sequences were tagged and removed. Sex chromosomes were identified by read coverage statistics. The curation process is documented at https://gitlab.com/wtsi-grit/rapid-curation (article in preparation).

***Evaluation of the final assembly***

The final assembly was post-processed and evaluated using the three Nextflow ( Di Tommaso *et al.*, 2017) DSL2 pipelines sanger-tol/readmapping ( Surana *et al.*, 2023a), sanger-tol/genomenote ( Surana *et al.*, 2023b), and sanger-tol/blobtoolkit ( Muffato *et al.*, 2024). The readmapping pipeline aligns the Hi-C reads using bwa-mem2 ( Vasimuddin *et al.*, 2019) and combines the alignment files with SAMtools ( Danecek *et al.*, 2021). The genomenote pipeline transforms the Hi-C alignments into a contact map with BEDTools ( Quinlan & Hall, 2010) and the Cooler tool suite ( Abdennur & Mirny, 2020). The contact map is visualised in HiGlass ( Kerpedjiev *et al.*, 2018). This pipeline also generates assembly statistics using the NCBI datasets report ( Sayers *et al.*, 2024), computes *k*-mer completeness and QV consensus quality values with FastK and MERQURY.FK, and runs BUSCO ( Manni *et al.*, 2021) to assess completeness.

The sanger-tol/blobtoolkit pipeline is a Nextflow port of the previous Snakemake Blobtoolkit pipeline ( Challis *et al.*, 2020). It aligns the PacBio reads in SAMtools and minimap2 ( Li, 2018) and generates coverage tracks for regions of fixed size. In parallel, it queries the GoaT database ( Challis *et al.*, 2023) to identify all matching BUSCO lineages to run BUSCO ( Manni *et al.*, 2021). For the three domain-level BUSCO lineages, the pipeline aligns the BUSCO genes to the UniProt Reference Proteomes database ( Bateman *et al.*, 2023) with DIAMOND ( Buchfink *et al.*, 2021) blastp. The genome is also split into chunks according to the density of the BUSCO genes from the closest taxonomically lineage, and each chunk is aligned to the UniProt Reference Proteomes database with DIAMOND blastx. Genome sequences without a hit are chunked with seqtk and aligned to the NT database with blastn ( Altschul *et al.*, 1990). The blobtools suite combines all these outputs into a blobdir for visualisation.

The genome assembly and evaluation pipelines were developed using nf-core tooling ( Ewels *et al.*, 2020) and MultiQC ( Ewels *et al.*, 2016), relying on the Conda package manager, the Bioconda initiative ( Grüning *et al.*, 2018), the Biocontainers infrastructure ( da Veiga Leprevost *et al.*, 2017), as well as the Docker ( Merkel, 2014) and Singularity ( Kurtzer *et al.*, 2017) containerisation solutions.

Table 4 contains a list of relevant software tool versions and sources.

**Table 4.** Software tools: versions and sources.

Software tool	Version	Source
BEDTools	2.30.0	https://github.com/arq5x/bedtools2
BLAST	2.14.0	ftp://ftp.ncbi.nlm.nih.gov/blast/executables/blast+/
BlobToolKit	4.3.7	https://github.com/blobtoolkit/blobtoolkit
BUSCO	5.4.3 and 5.5.0	https://gitlab.com/ezlab/busco
bwa-mem2	2.2.1	https://github.com/bwa-mem2/bwa-mem2
Cooler	0.8.11	https://github.com/open2c/cooler
DIAMOND	2.1.8	https://github.com/bbuchfink/diamond
fasta_windows	0.2.4	https://github.com/tolkit/fasta_windows
FastK	427104ea91c78c3b8b8b49f1a7d6b beaa869ba1c	https://github.com/thegenemyers/FASTK
Gfastats	1.3.6	https://github.com/vgl-hub/gfastats
GoaT CLI	0.2.5	https://github.com/genomehubs/goat-cli
Hifiasm	0.19.8-r603	https://github.com/chhylp123/hifiasm
HiGlass	44086069ee7d4d3f6f3f001256978 9ec138f42b84aa44357826c0b675 3eb28de	https://github.com/higlass/higlass
Merqury.FK	d00d98157618f4e8d1a9190026b19 b471055b22e	https://github.com/thegenemyers/MERQURY.FK
MitoHiFi	3	https://github.com/marcelauliano/MitoHiFi
MultiQC	1.14, 1.17, and 1.18	https://github.com/MultiQC/MultiQC
NCBI Datasets	15.12.0	https://github.com/ncbi/datasets
Nextflow	23.04.0-5857	https://github.com/nextflow-io/nextflow
PretextView	0.2	https://github.com/sanger-tol/PretextView
purge_dups	1.2.5	https://github.com/dfguan/purge_dups
samtools	1.16.1, 1.17, and 1.18	https://github.com/samtools/samtools
sanger-tol/ascc	-	https://github.com/sanger-tol/ascc
sanger-tol/genomenote	1.1.1	https://github.com/sanger-tol/genomenote
sanger-tol/readmapping	1.2.1	https://github.com/sanger-tol/readmapping
Seqtk	1.3	https://github.com/lh3/seqtk
Singularity	3.9.0	https://github.com/sylabs/singularity
TreeVal	1.0.0	https://github.com/sanger-tol/treeval
YaHS	1.2a.2	https://github.com/c-zhou/yahs

### Wellcome Sanger Institute – Legal and Governance

The materials that have contributed to this genome note have been supplied by a Darwin Tree of Life Partner. The submission of materials by a Darwin Tree of Life Partner is subject to the **‘Darwin Tree of Life Project Sampling Code of Practice’**, which can be found in full on the Darwin Tree of Life website here. By agreeing with and signing up to the Sampling Code of Practice, the Darwin Tree of Life Partner agrees they will meet the legal and ethical requirements and standards set out within this document in respect of all samples acquired for, and supplied to, the Darwin Tree of Life Project.

Further, the Wellcome Sanger Institute employs a process whereby due diligence is carried out proportionate to the nature of the materials themselves, and the circumstances under which they have been/are to be collected and provided for use. The purpose of this is to address and mitigate any potential legal and/or ethical implications of receipt and use of the materials as part of the research project, and to ensure that in doing so we align with best practice wherever possible. The overarching areas of consideration are:

•   Ethical review of provenance and sourcing of the material 

•   Legality of collection, transfer and use (national and international) 

Each transfer of samples is further undertaken according to a Research Collaboration Agreement or Material Transfer Agreement entered into by the Darwin Tree of Life Partner, Genome Research Limited (operating as the Wellcome Sanger Institute), and in some circumstances other Darwin Tree of Life collaborators.

## Data Availability

European Nucleotide Archive: *Anser brachyrhynchus* (pink-footed goose). Accession number PRJEB68284; https://identifiers.org/ena.embl/PRJEB68284 ( Wellcome Sanger Institute, 2024). The genome sequence is released openly for reuse. The *Anser brachyrhynchus* genome sequencing initiative is part of the Darwin Tree of Life (DToL) project. All raw sequence data and the assembly have been deposited in INSDC databases. The genome will be annotated using available RNA-Seq data and presented through the Ensembl pipeline at the European Bioinformatics Institute. Raw data and assembly accession identifiers are reported in Table 1 and Table 2.

## Author information

Members of the Natural History Museum Genome Acquisition Lab are listed here: https://doi.org/10.5281/zenodo.12159242.

Members of the Darwin Tree of Life Barcoding collective are listed here: https://doi.org/10.5281/zenodo.12158331

Members of the Wellcome Sanger Institute Tree of Life Management, Samples and Laboratory team are listed here: https://doi.org/10.5281/zenodo.12162482.

Members of Wellcome Sanger Institute Scientific Operations: Sequencing Operations are listed here: https://doi.org/10.5281/zenodo.12165051.

Members of the Wellcome Sanger Institute Tree of Life Core Informatics team are listed here: https://doi.org/10.5281/zenodo.12160324.

Members of the Tree of Life Core Informatics collective are listed here: https://doi.org/10.5281/zenodo.12205391.

Members of the Darwin Tree of Life Consortium are listed here: https://doi.org/10.5281/zenodo.4783558.
