# Supplementary material for: The genome sequence of the pink-footed goose, Anser brachyrhynchus Baillon, 1834
Source: Wellcome Open Res. 2024 Oct 18;9:613. [Version 1] doi: 10.12688/wellcomeopenres.23194.1 (PMC11803379; doi:10.12688/wellcomeopenres.23194.1)
Supplement: Peer review history

# Reviewer report: Reviewer response for version 1

**DOI:** 10.21956/wellcomeopenres.25544.r109145

**Published:** 2024-12-12

**Authors:** Jian Wang, Hongchang Zhao

**Affiliations:** 1 Jiangsu Agri-animal Husbandry Vocational College, Taizhou, China

Overall, I am quite satisfied with the quality and presentation of this paper. The genome assembly of *Anser brachyrhynchus* is conducted with a clear and methodologically sound approach, providing valuable insights into the genomic structure of this species. The authors have detailed the assembly process thoroughly, and the use of 41 chromosomal pseudomolecules, including the W and Z sex chromosomes, is commendable. The assembly spans a substantial 1,287.30 megabases, which offers a robust foundation for further genetic studies in this species.

 The characterization of the genomic assembly is particularly impressive. The authors have carefully described key assembly metrics, offering a detailed view of the quality and completeness of the genome. The mitochondrial genome assembly is also a valuable addition, providing further depth to the study.

 One area for improvement would be the inclusion of genome annotation information, which would provide additional insights into the functional aspects of the genome. The incorporation of gene annotations could enhance the utility of the dataset for downstream analyses, such as functional genomics and comparative genomics.

 In summary, this paper presents a significant contribution to the field of avian genomics, with a well-executed assembly process and comprehensive assembly statistics. The study lays a strong foundation for future research in this area, though the addition of genome annotation would make the data even more impactful.

Are sufficient details of methods and materials provided to allow replication by others?

Yes

Is the rationale for creating the dataset(s) clearly described?

Yes

Are the datasets clearly presented in a useable and accessible format?

Yes

Are the protocols appropriate and is the work technically sound?

Yes

Reviewer Expertise:

My area of expertise lies in the functional gene discovery and exploration in livestock and poultry

We confirm that we have read this submission and believe that we have an appropriate level of expertise to confirm that it is of an acceptable scientific standard.

# Reviewer report: Reviewer response for version 1

**DOI:** 10.21956/wellcomeopenres.25544.r106425

**Published:** 2024-11-01

**Authors:** Guangliang Gao

**Affiliations:** 1 Chongqing Academy of Animal Science, Chongqing, China

I am writing to provide my review of the manuscript titled “The genome sequence of the pink-footed goose, *Anser brachyrhynchus *Baillon, 1834.” After carefully evaluating the content and methodology presented in the paper, I am pleased to report that I have no further modification suggestions. The authors have conducted a comprehensive analysis that significantly contributes to our understanding of the genomic features of the pink-footed goose. The methodology is robust, and the results are presented clearly and effectively. The findings have important implications for the study of avian genetics and conservation.

Are sufficient details of methods and materials provided to allow replication by others?

Yes

Is the rationale for creating the dataset(s) clearly described?

Yes

Are the datasets clearly presented in a useable and accessible format?

Yes

Are the protocols appropriate and is the work technically sound?

Yes

Reviewer Expertise:

Our work primarily focuses on genetic breeding of domestic geese and genomic studies of the Anserinae subfamily, which includes wild graylag geese, bean geese, and other wild geese

I confirm that I have read this submission and believe that I have an appropriate level of expertise to confirm that it is of an acceptable scientific standard.
